# Excess mortality in Europe coincides with peaks of COVID-19, influenza and respiratory syncytial virus (RSV), November 2023 to February 2024

**DOI:** 10.2807/1560-7917.ES.2024.29.15.2400178

**Published:** 2024-04-11

**Authors:** Sarah K Nørgaard, Jens Nielsen, Anne Christine Nordholm, Lukas Richter, Alena Chalupka, Natalia Bustos Sierra, Toon Braeye, Maria Athanasiadou, Theodore Lytras, Gleb Denissov, Oskari Luomala, Anne Fouillet, Isabelle Pontais, Matthias an der Heiden, Benedikt Zacher, Alina Weigel, Ivo Foppa, Kassiani Gkolfinopoulou, Ioannis Panagoulias, Anna Paldy, Tibor Malnasi, Lisa Domegan, Eva Kelly, Naama Rotem, Oksana Rakhlin, Francesca K de'Donato, Chiara Di Blasi, Patrick Hoffmann, Telma Velez, Kathleen England, Neville Calleja, Liselotte van Asten, Femke Jongenotter, Ana Paula Rodrigues, Susana Silva, Petra Klepac, Diana Gomez-Barroso, Inmaculada Leon Gomez, Ilias Galanis, Ahmed Farah, Rolf Weitkunat, Katarina Fehst, Nick Andrews, Tom Clare, Declan T Bradley, Mark G O'Doherty, Naoma William, Mark Hamilton, Bolette Søborg, Tyra G Krause, Nick Bundle, Lasse S Vestergaard

**Affiliations:** 1 Department of Infectious Disease Epidemiology and Prevention, Statens Serum Institut, Copenhagen, Denmark; 2 Austrian Agency for Health and Food Safety, Vienna, Austria; 3 Sciensano, Brussels, Belgium; 4 Health Monitoring Unit, Cyprus Ministry of Health, Nicosia, Cyprus; 5 School of Medicine, European University Cyprus, Nicosia, Cyprus; 6 National Institute for Health Development, Tallinn, Estonia; 7 Finnish Institute for Health and Welfare (THL), Helsinki, Finland; 8 Santé publique France, Saint-Maurice, France; 9 Robert Koch Institute, Berlin, Germany; 10 Hessisches Landesamt für Gesundheit und Pflege, Dillenburg, Germany; 11 Hellenic National Public Health Organization, Athens, Greece; 12 National Center for Public Health and Pharmacy, Budapest, Hungary; 13 Health-Service Executive - Health Protection Surveillance Centre, Dublin, Ireland; 14 Central Bureau of Statistics, Jerusalem, Israel; 15 Department of Epidemiology Lazio Regional Health System - ASL Roma 1, Rome, Italy; 16 Health Directorate, Luxembourg, Luxembourg; 17 Directorate for Health Information and Research, Pieta, Malta; 18 Centre for Infectious Disease Control Netherlands, National Institute for Public Health and the Environment (RIVM), Bilthoven, The Netherlands; 19 Department of Epidemiology, Instituto Nacional de Saúde Dr. Ricardo Jorge, Lisbon, Portugal; 20 Communicable Diseases Centre, National Institute of Public Health, Ljubljana, Slovenia; 21 National Centre of Epidemiology, CIBER Epidemiología y Salud Pública (CIBERESP), Carlos III Health Institute, Madrid, Spain; 22 Public Health Agency of Sweden, Stockholm, Sweden; 23 Federal Statistical Office, Neuchâtel, Switzerland; 24 UK Health Security Agency, London, United Kingdom; 25 Public Health Agency, Northern Ireland, United Kingdom; 26 Public Health Scotland, Glasgow, United Kingdom; 27 European Centre for Disease Prevention and Control (ECDC), Stockholm, Sweden

**Keywords:** Excess mortality, EuroMOMO, surveillance, respiratory infections

## Abstract

Since the end of November 2023, the European Mortality Monitoring Network (EuroMOMO) has observed excess mortality in Europe. During weeks 48 2023–6 2024, preliminary results show a substantially increased rate of 95.3 (95% CI:  91.7–98.9) excess all-cause deaths per 100,000 person-years for all ages. This excess mortality is seen in adults aged 45 years and older, and coincides with widespread presence of COVID-19, influenza and respiratory syncytial virus (RSV) observed in many European countries during the 2023/24 winter season.

Excess mortality has typically been observed during the winter season in Europe, coinciding with epidemics of seasonal influenza, respiratory syncytial virus (RSV) and other respiratory infections [1,2]. However, this typical seasonal pattern was largely disrupted during the COVID-19 pandemic from 2020 to 2022. Several periods of excess mortality were observed ‘out of season’ and related to consecutive waves of severe acute respiratory syndrome coronavirus 2 (SARS-CoV-2) causing COVID-19 [3-7], while common respiratory infections were absent because of implementation of non-pharmacological interventions (NPIs) [8,9]. When NPIs were gradually lifted, respiratory infections returned, though displaying some atypical seasonality during 2021 and 2022 [10-14]. In the winter season of 2023/24, excess mortality was again observed in Europe. Here, we present pooled estimates of excess all-cause mortality together with estimates of test positivity of COVID-19, influenza and RSV across 25 countries or subnational areas in the European Mortality Monitoring Network (EuroMOMO).

## EuroMOMO’s estimations of excess all-cause mortality

The EuroMOMO network has monitored the weekly level of excess all-cause mortality in the participating European countries or subnational areas and at the European level based on pooled, country-stratified analyses since 2009. The framework and methodology of EuroMOMO has been described previously [15-17]. 

In this report, ‘Europe’ broadly refers to the 25 countries participating in the EuroMOMO network, predominantly consisting of Western European countries and covering in total ca 390 million people. The following 25 participating European countries or subnational areas contributed with weekly all-cause mortality data to the analyses presented: Austria, Belgium, Cyprus, Denmark, Estonia, Finland, France, Germany, Greece, Hungary, Ireland, Israel, Italy, Luxembourg, Malta, the Netherlands, Portugal, Slovenia, Spain, Sweden, Switzerland and the United Kingdom (UK) (England, Northern Ireland, Scotland, Wales). Participating countries provide national data, with the exception of Italy that reports only data from cities with populations over 100,000 inhabitants. Data on population size were extracted from Eurostat [18], except for Israel, for which the population was retrieved from the Health and Vital Statistics Sector, Central Bureau of Statistics. 

Weekly numbers of excess all-cause deaths were estimated for the total population (all ages) and for age groups (0–14 years, 15–44 years, 45–64 years, 65–74 years, 75–84 years and ≥ 85 years) from week 41 2023 up to and including week 12 2024, based on data collected up to week 13 2024, i.e. including data up to the end of week 12. Mortality rates per 100,000 person-years were calculated, as well as the number of excess deaths as a percentage of the expected number of deaths. Furthermore, we calculated mortality rates for the period week 48 2023 to week 6 2024 where the EuroMOMO network observed a substantially increased excess mortality. Z-scores were used to display the variation of excess mortality. ‘Increased mortality’ was defined when the z-score exceeded 2 and ‘substantially increased mortality’ when it exceeded 4. 

Because of the long period of unexpected increased mortality in Europe attributable to the COVID-19 pandemic in the period from 2020 to 2022, mortality data from these 3 years were omitted from the estimation of expected mortality. The overall pooled mortality estimates were not age-stratified.

## Pooled estimates of excess all-cause mortality in winter 2023/24

**Figure 1:**
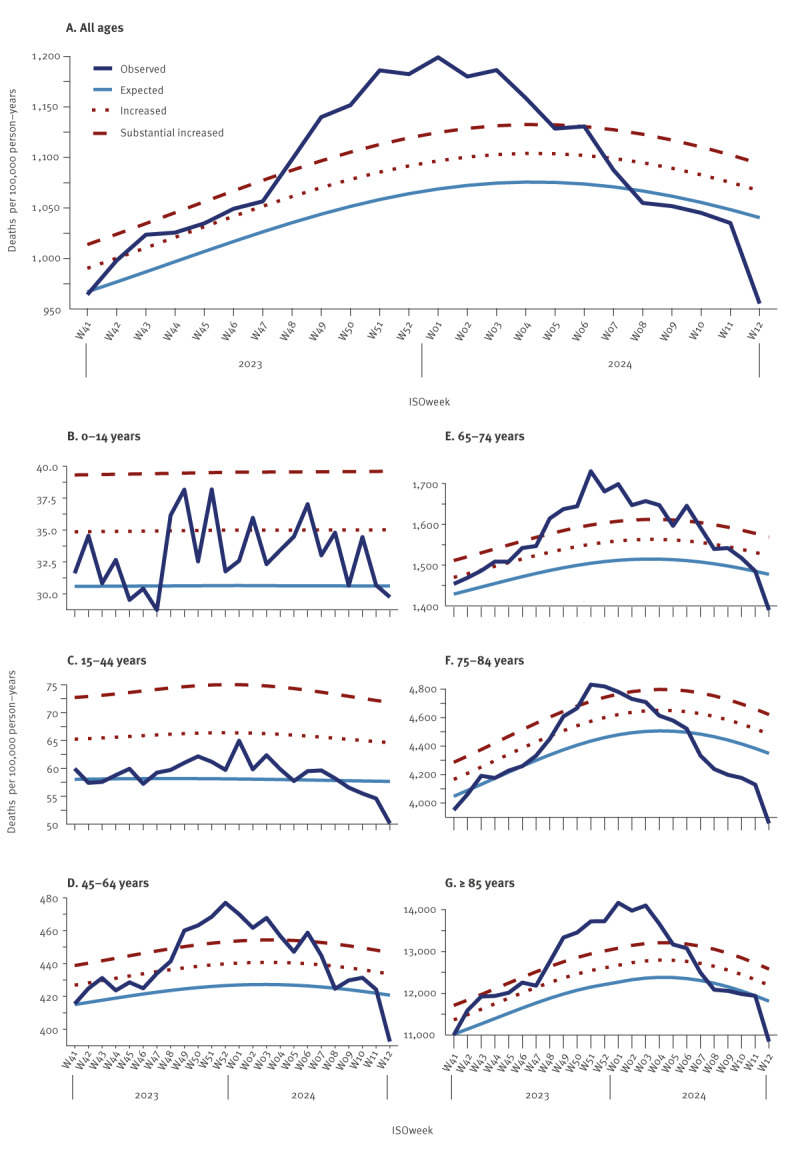
EuroMOMO pooled estimates and observed numbers of all-cause mortality per 100,000 person-years for (A) all ages and (B–G) by age group, 25 participating countries or subnational areas^a^, week 41 2023–week 12 2024

From week 43 2023 onwards, an increasing trend in excess deaths was observed, with pooled estimates of all-cause deaths exceeding 2 z-scores above the expected level, and from week 48 2023, a continuous substantially increased excess mortality was observed (Figure 1). The increased mortality was primarily seen among individuals aged 45 years or older, while no consistent excess mortality was observed among individuals younger than 45 years.

EuroMOMO: European mortality monitoring network.

^a^ Detailed information about countries and subnational areas participating in EuroMOMO can be found on the EuroMOMO website [16].

Estimated expected all-cause mortality is shown together with the observed number of deaths per 100,000 person-years, based on data received by the EuroMOMO hub in week 13 2024. The observed number of deaths per 100,000 person-years is adjusted for delay in registration in the most recent weeks. Note that the y-axis is age-group dependent. An interactive graphic platform of previous periods can be accessed on the EuroMOMO network's website (Euromomo.eu) for historical mortality data. ‘Increased mortality’ is defined as a z-score exceeding 2 and ‘substantially increased mortality’ is defined as a z-score exceeding 4.

The pooled estimates showed a peak number of 9,503 (95% confidence interval (CI): 7,542–11,481) excess all-cause deaths for all ages in week 51 2023, corresponding to a mortality level 12.1% (95% CI: 9.6–14.6) higher than expected. The peak in excess mortality rate was the highest among adults aged 85 years and above, showing 1,571.0 (95% CI: 1,188.1–1,957.9) excess deaths per 100,000 person-years (4,330 excess deaths, 95% CI: 3,275–5,396), or 12.9% (95% CI: 9.8–16.1) higher than expected.

Considering the peak of substantial excess mortality from week 48 2023 to week 6 2024, we found a rate of 95.3 (95% CI: 91.7–98.9) excess all-cause deaths per 100,000 person-years for all ages (Table).

**Table:** EuroMOMO pooled estimates of excess all-cause mortality for the period with substantial elevated mortality by age group, 25 participating countries or subnational areas^a^, week 48 2023–week 6 2024

Age group (years)	At-risk individuals (x 100,000) by week 41/2023	Observed person-years (x 100,000)	Observed all-cause deaths	Expected all-cause deaths	Estimated excess all-cause deaths		Estimated excess all-cause deaths per 100,000 person-years	
	n	n	n	n	n	95% CI	n	95% CI
All ages	3,877.7	818.0	947,570	869,643	77,926	74,980–80,910	95.3	91.7–98.9
0–14	587.1	123.0	4,280	3,769	511	433–592	4.1	3.5–4.8
15–44	1,411.4	297,6	18,071	17,283	788	552–1051	2.6	1.9–3.5
45–64	1,053.7	222,2	102,438	94,679	7,759	7,387–8,138	34.9	33.2–36.6
65–74	416.0	175,3	823,024	756,480	66,544	63,800–69,325	379.7	364–395.5
75–84	269.4	87,9	145,413	132,414	12,999	12,440–13,566	147.9	141.5–154.4
≥ 85	139.0	57.0	265,925	253,946	11,979	11,137–12,840	210.1	195.4–225.2

CI: confidence interval; EuroMOMO: European mortality monitoring network.

^a^ Detailed information about countries and subnational areas participating in EuroMOMO can be found on the EuroMOMO website [16].

## Country estimates of excess mortality in winter 2023/24

**Figure 2:**
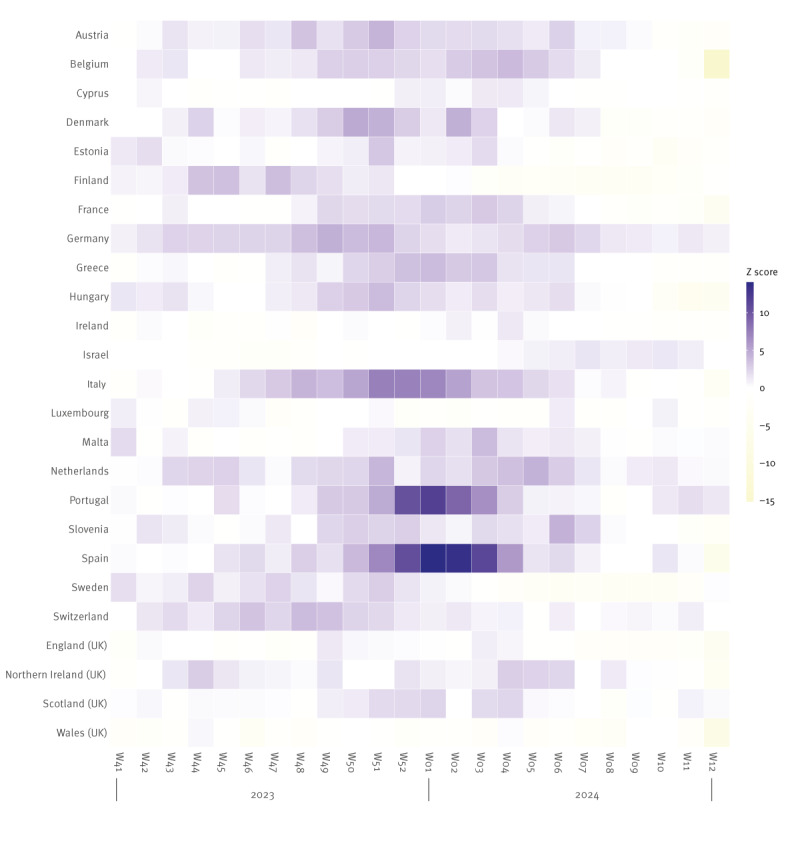
Estimated z-scores of excess all-cause mortalities by ISO week, 25 participating countries or subnational areas^a^, week 41 2023–week 12 2024

^a^ Detailed information about countries and subnational areas participating in EuroMOMO can be found on the EuroMOMO website [16].

In week 43 2023, where the pooled excess all-cause mortality for all ages exceeded 2 z-scores for the first time, Germany, the Netherlands and Switzerland reported increased mortality, followed by Denmark, Finland, Sweden and Northern Ireland (UK) in week 44 2023 (Figure 2).

When the pooled all-cause excess mortality for all ages increased to a substantial level, i.e. by week 48 2023, the following six participating countries reported increased all-cause mortality: Denmark, Finland, Germany, the Netherlands, Sweden and Northern Ireland (UK).

Overall, through the period from week 48 2023 to week 6 2024, increased all-cause mortality was reported in 19 of the 25 countries or subnational areas in the EuroMOMO network. Cyprus, Ireland, Israel, Luxembourg, England (UK) and Wales (UK) did not report elevated all-cause mortality for all ages during this period.

## Epidemics of COVID-19, influenza and RSV infections in winter 2023/24

**Figure 3:**
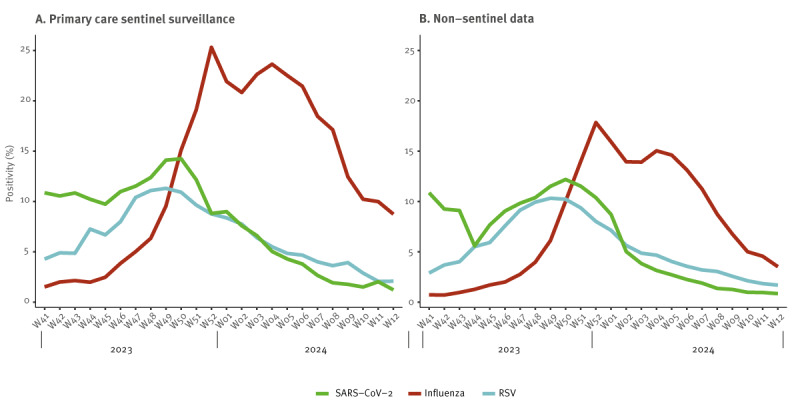
Pathogen-specific test positivity of SARS-CoV-2, influenza and RSV for all ages based on (A) primary care sentinel data and (B) non-sentinel data submitted to TESSy, 25 countries^a^, week 41 2023–week 12 2024

RSV: respiratory syncytial virus; SARS-CoV-2: severe acute respiratory syndrome coronavirus 2; The European Surveillance System (TESSy).

^a^ Included countries:

To identify possible explanations for the high level of excess mortality observed, we explored the level of circulation of the three main respiratory viral infections and occurrence of associated diseases in the EuroMOMO countries in the 2023/24 winter season: COVID-19, influenza and RSV infections. We analysed weekly national data from primary care sentinel surveillance systems and non-sentinel data reported to The European Surveillance System (TESSy [19]) and published in the European Respiratory Virus Surveillance Summary (ERVISS [20]) at the European Centre for Disease Prevention and Control (ECDC). We estimated pathogen-specific test positivity by dividing the sum of the weekly detections by the tests reported per pathogen summed across each of the participating countries for which data were available (Figure 3). Data for a given country-pathogen-week were included only if both tests and detections were reported and the number of tests was greater than zero. Patterns were similar in the sentinel and non-sentinel surveillance systems. 

**A. Primary care sentinel data - SARS-CoV-2: **Belgium, Denmark, Estonia, Finland, France, Germany, Greece, Hungary, Ireland, Israel, Italy, Luxembourg, Malta, Netherlands, Portugal, Slovenia, Spain, Sweden, Switzerland, England (UK), Scotland (UK), Wales (UK). **Influenza: **Austria, Belgium, Denmark, Estonia, Finland, France, Germany, Greece, Hungary, Ireland, Israel, Italy, Luxembourg, Malta, Netherlands, Portugal, Slovenia, Spain, Sweden, Switzerland, Northern Ireland (UK), Scotland (UK), Wales (UK). **RSV:** Belgium, Denmark, Estonia, Finland, France, Germany, Greece, Hungary, Ireland, Israel, Luxembourg, Netherlands, Portugal, Slovenia, Spain, Switzerland, Northern Ireland (UK), Scotland (UK), Wales (UK).

**B. Non-sentinel data from TESSy - SARS-CoV-2**: Belgium, Cyprus, Denmark, Estonia, Greece, Hungary, Ireland, Italy, Luxembourg, Netherlands, Portugal, Slovenia, Sweden, England (UK), Northern Ireland (UK), Scotland (UK). **Influenza: **Austria, Denmark, Estonia, Finland, France, Germany, Greece, Hungary, Ireland, Israel, Italy, Malta, Portugal, Slovenia, Spain, Sweden, Northern Ireland (UK), Scotland (UK), Wales (UK). **RSV: **Denmark, Estonia, Finland, France, Germany, Greece, Hungary, Ireland, Israel, Malta, Portugal, Slovenia, Spain, Sweden, Northern Ireland (UK), Scotland (UK), Wales (UK).

For both A and B, data from Italy include the entire country.

COVID-19 was the most common disease caused by the respiratory viruses considered at the start of the study period, having increased steadily since late summer 2023 [20]. SARS-CoV-2 test positivity peaked in week 50 2023, after which it declined rapidly to low levels during January and February 2024. Test positivity for RSV peaked at a similar time but declined more steadily than SARS-CoV-2 during January and February 2024. Influenza showed a fairly typical pattern with a sharp increase from week 48 2024, reaching peak levels in late December 2023–January 2024 before declining steadily.

## Discussion

After several years of some unusual mortality patterns observed during the COVID-19 pandemic, the mortality in Europe overall now in the winter of 2023/24 appears to have returned to a pattern more similar to that observed before the COVID-19 pandemic. The EuroMOMO network observed a notable increase in all-cause mortality from week 43 2023 to week 6 2024. This excess mortality was primarily observed among individuals aged 45 years or older, but with a particularly high rate of excess mortality in adults aged 85 years and above, although with some variation between countries.

While we cannot attribute the current excess mortality to specific causes, given that many adults are hospitalised with either COVID-19, influenza or RSV infections, it may be presumed that these respiratory diseases contributed significantly to the elevated mortality. Their individual contribution to excess mortality will depend on multiple factors, including the virulence of the circulating viruses, severity of disease caused by them, the population immunity landscape and the age profile of people infected. However, high coverage of COVID-19 vaccines with high effectiveness in some countries could also have contributed to reducing the mortality in the winter of 2023/24. Thus far, SARS-CoV-2, the virus causing COVID-19, appears to be present year-round; therefore, it is possible that there could be occasional surges in excess mortality outside of the winter season particularly in older individuals, driven by new SARS-CoV-2 variants.

It should be emphasised that our presented estimates of excess mortality are limited to available data reported from the 25 countries participating in the EuroMOMO network, and therefore are not representative of the entire population in the World Health Organization (WHO) European Region. Furthermore, the number of deaths shown for the 3 most recent weeks should be interpreted with some caution, as our applied adjustments for the delay in registration may be imprecise.

When estimating expected mortality, another factor to consider is the role of ageing European population. The effect of population growth is considered by using a linear trend in the model applied to estimate expected mortality; however, if this population growth occurs at an exponential rate, this linear trend may not fully capture the effect of population growth on mortality. This could be a concern for the age group of 85 years and older.

Despite the mentioned limitations, our report shows again how simple, consistent and coordinated weekly monitoring of excess all-cause mortality serves as an important and timely tool for public health [21], e.g. informing society about the severity and impact of current threats such as COVID-19 and other common seasonal respiratory epidemics.

## Conclusion

In the winter season 2023/24, EuroMOMO has observed some substantially increased mortality in Europe in a typical temporal pre-COVID-19 pandemic pattern, which have coincided with epidemic peaks of COVID-19, influenza and RSV infections in many of the participating countries. All-cause mortality is an important proxy measure of the severity and mortality impact of common seasonal respiratory infections in the population, and weekly monitoring of excess all-cause mortality remains an important and timely tool for public health.
